# Plant Metabolomics: The Future of Anticancer Drug Discovery

**DOI:** 10.3390/ph17101307

**Published:** 2024-09-30

**Authors:** Ranin Dabbousy, Mohamad Rima, Rabih Roufayel, Mohamad Rahal, Christian Legros, Jean-Marc Sabatier, Ziad Fajloun

**Affiliations:** 1 Laboratory of Applied Biotechnology (LBA3B), Department of Cell Culture, Azm Center for Research in Biotechnology and Its Applications, EDST, Lebanese University, Tripoli 1300, Lebanon; ranindabbousy.rd@gmail.com; 2 Department of Natural Sciences, Lebanese American University, Byblos P.O. Box 36, Lebanon; mohamad.rima@lau.edu.lb; 3 College of Engineering and Technology, American University of the Middle East, Egaila 54200, Kuwait; rabih.roufayel@aum.edu.kw; 4 School of Pharmacy, Lebanese International University, Beirut 146404, Lebanon; mohamad.rahal@liu.edu.lb; 5 INSERM, CNRS, MITOVASC, Equipe CarME, SFR ICAT, Faculty of Medicine, University Angers, 49000 Angers, France; christian.legros@univ-angers.fr; 6 CNRS, INP, Inst Neurophysiopathol, Aix-Marseille Université, 13385 Marseille, France; 7 Department of Biology, Faculty of Sciences 3, Campus Michel Slayman Ras Maska, Lebanese University, Tripoli 1352, Lebanon

**Keywords:** metabolomics, anticancer drugs, medicinal plants, drug discovery, bioactive metabolites, plant metabolomics, promising tool

## Abstract

Drug development from medicinal plants constitutes an important strategy for finding natural anticancer therapies. While several plant secondary metabolites with potential antitumor activities have been identified, well-defined mechanisms of action remained uncovered. In fact, studies of medicinal plants have often focused on the genome, transcriptome, and proteome, dismissing the relevance of the metabolome for discovering effective plant-based drugs. Metabolomics has gained huge interest in cancer research as it facilitates the identification of potential anticancer metabolites and uncovers the metabolomic alterations that occur in cancer cells in response to treatment. This holds great promise for investigating the mode of action of target metabolites. Although metabolomics has made significant contributions to drug discovery, research in this area is still ongoing. In this review, we emphasize the significance of plant metabolomics in anticancer research, which continues to be a potential technique for the development of anticancer drugs in spite of all the challenges encountered. As well, we provide insights into the essential elements required for performing effective metabolomics analyses.

## 1. Introduction

Natural extracts have always been a rich source of valuable pharmaceutical molecules [1,2]. Medicinal plants, in particular, are a major source of natural extracts that are extensively used in drug discovery [3,4]. In fact, most of the anticancer drugs approved by the United States Food and Drug Administration (US FDA) are mainly produced from templates of plant metabolites or their derivatives [5]. In this context, it was reported that a significant number of recently discovered cancer drugs contain natural constituents [6]. Among the most well-known plant-based anticancer drugs are camptothecin derivatives [7], taxol (paclitaxel) [8], and vinca alkaloids (vinblastine, vincristine) [9]. Plants contain a complex mixture of active substances and phytochemicals that are responsible for their biological activity [10]. Mainly, secondary metabolites having important pharmaceutical effects on multiple signaling pathways and molecular targets [11,12] are the main substances used in plant-derived drugs [13]. In fact, these compounds act at the cellular or the molecular level [14,15] by exhibiting antioxidant, antiinflammatory, antitumor, and anticarcinogenic effects [16]. Tannins, terpenoids, lignans, phenolic acids, quinones, flavonoids, alkaloids, coumarins, catechins, and isocatechins are among the metabolites that have a significant role in cancer treatment [17]. Although many studies focusing on compounds with anticancer properties from various medicinal plants have been reported [18,19], further research is still required to alleviate the challenges facing the medicinal plants pharmaceutical sector. These challenges include the scarcity of secondary metabolites [20], which can seriously hinder metabolite identification from the step of isolation to the drug development stage. The toxicity of medicinal plants also constitutes a major problem, as it can cause adverse effects in patients [21]. However, the comprehensive understanding of the mode of action of anticancer metabolites remains the main challenge. On the other hand, the heterogenicity of tumor cells and population polymorphism [22], in addition to their acquired resistance to anticancer treatments [23], make the situation more challenging. Even though, the anticancer activity of plant metabolites has been confirmed, new strategies are still required to depict the mechanism of action of these compounds. Therefore, it is crucial to elucidate the interactions between bioactive plant compounds and their targets, in order to understand their therapeutic effects [24]. Metabolomics is the inclusive study of all the metabolites found in a biological system, including carbohydrates, amino acids, lipids, organic acids [25], aldehydes [26], steroids [27], vitamins [28], polyphenols [29], hormones, and signaling molecules [30]. Over the past few years, metabolomics has stood out as a powerful tool for identifying novel therapeutic targets, opening up new routes for therapeutic approaches, especially for cancer [31]. Besides being a simple and effective tool [32], metabolomics enables the identification of the variations affecting the various metabolic pathways [33], the investigation of drug efficacy and toxicity [34], and the discovery of unique biomarkers [35]. Metabolomics also permits to identify the modifications affecting the endogenous and exogeneous metabolites of biological systems [36,37,38]. Multiple studies have applied metabolomics to medicinal plant research, some of which are nicely reviewed in [39]. Accordingly, the use of metabolomics has led to major findings, such as the elucidation of the anticancer mechanisms of plant metabolites [40], the identification and the validation of metabolic biomarkers for cancer diagnosis [41], and the investigation of tumor metabolic reprogramming, in addition to other applications in drug oncology [42]. The integration of genomics, transcriptomics, proteomics, and metabolomics has helped to achieve a comprehensive picture of bioactive chemicals of plant origin [43]. In this review, we focus on the contributions made by metabolomics studies to support plant-based anticancer drug discovery, and we emphasize its role in tackling the challenges associated to this field. In addition, we highlight how advances in metabolomics have enhanced our understanding of cancer heterogenicity among patients, leading to improved diagnosis. We also discuss the difficulties associated with metabolomics studies, and we provide some solutions. indeed, numerous studies exploring plant metabolomics and its relation to cancer research can be found in the literature including the specific book chapter [44] and the review by Yani Magfiroh, which examined 28 medicinal plants, and provided the analytical techniques employed, the biomarkers found, and the major discoveries made [39]. However, in this review we highlight the key points that should be considered and the errors to be avoided in order to perform an effective metabolomics study directed to plant cancer research. Therefore, our review provides a comprehensive understanding of cancer-related metabolomics, encompassing its advantages and limitations, in order to establish a well-designed metabolomics process. 

## 2. Plant Metabolomics as a Key Tool for Anticancer Studies

Cancer therapies can be divided into conventional (chemotherapy, radiotherapy, and surgical) and modern therapies (hormone therapy, stem cell therapies, anti-angiogenic, immunotherapy, and dendritic cell-based immunotherapy) [45]. Several therapies have severe side effects [46,47], and patients often exhibit resistance [48], or show poor response to these therapies [49,50]. Consequently, it is essential to develop treatments for cancer that are both safer and more effective, while minimizing side effects. Many studies have been conducted over the years aiming to find new anticancer therapies, including those based on medicinal plants [51]. Plant-based treatments are promising, where, plant bioactive compounds act on different pathways involved in cancer tumorigenesis [52]. But, the process of identifying bioactive compounds from plant extracts is long and challenging [53]. Until recently, the available methods were time consuming and based on bioactivity-guided fractionation [54,55]. Mainly, the conventional methods for the identification of anticancer phytochemicals typically start with crude plant extraction by different solvents, then plant extracts are separated using different fractionation methods and the bioactive compounds are purified by various purification techniques [56]. Notably, the complex composition of plant extracts is a major constraint scientists face when searching for effective and safe plant treatments. Plant extracts and their fractions are then screened using different bioassays for determining their antiproliferative and cytotoxicity effects against cancer cells [54,57]. Mechanism-based screening methods are also used for screening anticancer plant-derived compounds by focusing on the pathways that are activated. [58,59]. Finally, in vivo trials involving animals and humans allow for the validation of the extracts’ efficacy [60,61]. Nevertheless, most of these studies lack a clear investigation of the anticancer mechanism of action exhibited by the bioactive metabolites [62,63]. Therefore, developing new methods that provide a better understanding of the mode of action of plant metabolites is crucial. This helps in the discovery of more targeted drugs, which in its turn lead to the development of more effective treatments with reduced non-desirable side effects. In fact, researchers have high expectations for the use of metabolomics in anticancer drug discovery due to a number of reasons. Metabolomics plays a key role in determining the mechanism of action of plant extracts as it identifies the altered metabolites and their underlying pathways [64]. For instance, cancer triggers the overexpression of several cell components, such as transporters and enzymes, which constitute good targets for anticancer drugs’ identification [65,66]. Thus, the anticancer activity of natural products should be ascertained through the metabolic fingerprinting and footprinting of cancer cells both prior to and following treatment [67]. Another great feature of metabolomics is that it permits to identify all the metabolic alterations that occur in the body following treatment. That is, it allows to measure both the intracellular [68] and the extracellular metabolites (exometabolomic) that are either released by cells into the extracellular medium or are a result of the different biochemical transformations occurring in the organism [69]. Thus, a comprehensive understanding of the metabolome provides a better picture of the altered signaling pathways and the cellular biology of cells, opening up new routes for drug optimization. Numerous studies have employed metabolomics to identify anticancer metabolites from medicinal plants. Herein, we highlight some of the major findings obtained by these studies. For instance, the secondary metabolites from *Ammi visnaga* L plant roots extract were identified using metabolomics based on high-performance liquid chromatography–heated electrospray ionization–high-resolution mass spectrometry (HPLC–HESI–HRMS), concurrently with assessing their antiproliferative activity. *Ammi visnaga* L plant roots extract exhibited antiproliferative effects against different types of cancer cells. Four major compounds, including Junipediol A 4-O glucoside (**1**), Junipediol A 8-O-glucoside (**2**), acacetin (**3**), and apiumetin-O-glucoside (**4**), were characterized. These metabolites showed an affinity for binding to the epidermal growth factor receptor (EGFR) tyrosine kinase, suggesting them as EGFR inhibitors, which was proposed as one of the molecular mechanisms of the antiproliferative properties of this plant [70]. A high-performance liquid chromatography–mass spectrometry (HPLC-MS) and nuclear magnetic resonance (NMR) spectroscopy-based metabolomics approach was employed to identify the bioactive compounds of two chamomile varieties, the Jordanian and European chamomiles, and to distinguish between these metabolites based on their anticancer and antioxidant activities. Among the isolated metabolites, two compounds from the European samples, chrysosplenetin (**4**) and apigenin (**5**), showed anticancer activity against a breast cancer cell line (ZR-75 cells), with IC_50_ values of 21.07 µg/mL and 22.55 µg/mL, respectively. Also, the metabolomics analysis revealed that the European chamomile produced more classes of metabolites with respect to the other plant variety, which was grown under distinct environmental conditions [71]. A gas chromatography–mass spectrometry (GC-MS) and liquid chromatography coupled to tandem mass spectrometry (LC-MS) untargeted metabolomics analysis was used to investigate the stigma ethanol extract of *Crocus cancellatus*. The evaluation of the anticancer properties of this extract showed antiproliferative activity against human breast cancer cell lines (MDA-MB-231 and MCF-7). This activity was suggested to be related to the action of the cytotoxic metabolites, including crocin (**6**), crocetin (**7**), picrocrocin (**8**), and safranal (**9**). This work also presented a potent approach based on the selection of specific LC-MS regions, called regions of interest (ROI), in addition to a multivariate curve resolution (MCRALS), which was used for the analysis of the LC-MS data. Moreover, it allowed to differentiate between the metabolites found in the extracts of two plants of the same species, *Crocus sativus* and. *Crocus cancellatus,* and helped in the evaluation of concentrations differences between the plant samples [72]. In addition, a widely targeted metabolomics analysis based on liquid chromatography with tandem mass spectrometry (LC-MS/MS) analysis allowed to determine the metabolite variability in different colors of carnation flowers. The key metabolites with different antioxidant and anticancer activities were highly present in the purple flower in comparison to the other colored flowers. Among the major compounds contributing to its antioxidant and anticancer effects, 2′-deoxyguanosine, 6-hydroxykaempferol-3,6-*O*-diglucoside, 6-hydroxykaempferol-7-*O*-glucoside, and quercetin-3-*O*-sophoroside were mentioned. 2′-deoxyguanosine was found to have anticancer activity against human cancer cell lines (A549 and U2OS). Interestingly, synergistic effects were also found between 2′-deoxyguanosine (**12**) and 6-hydroxykaempferol-3,6-*O*-diglucoside (**13**) or quercetin-3-*O*-sophoroside (**14**), which increased the anticancer activity of 2′-deoxyguanosine [73]. A rapid NMR-based metabolomics approach integrated with biological assays was used to identify the secondary metabolites in fourteen Fabaceae species of Mediterranean vegetation. Parallel biological assays helped to determine the antiproliferative activity against human colorectal cancer cell lines. Cycloartane glycoside (**15**) and protodioscin derivative (**16**) were found to have antiproliferative effects on colon cancer cell lines [74]. An approach based on the combination of the total phenolic and flavonoid contents, 3-(4,5-dimethylthiazol-2-yl)-2,5-diphenyltetrazolium bromide (MTT) assays, and LC-MS/MS-based metabolomics analysis helped to determine the phytochemical profile of *Glochidion velutinum*. The results revealed four compounds, epigallocatechin gallate (**17**), isovitexin (**18**), ellagic acid (**19**), and rutin (**20**), possessing anticancer activity against both prostate and breast cancer cell lines [75]. Phytochemical profiling of *Citrus aurantifolia* plant was performed using a combination of liquid chromatography–quadrupole time-of-flight tandem mass spectrometry (LC-QTOF/MS) and gas chromatography–high-resolution mass spectrometry (GC-HRMS). The anticancer activities of its ethanolic extract against liver cancer cells was determined using MTT assay that showed two compounds, hesperidin (**21**) and limonin (**22**) possessing an average IC_50_ of 165.615 and 188.073 µg/mL, respectively. A synergistic effect of limonin and hesperidin on apoptosis induction was revealed [76]. A 1H NMR-based metabolomics method allowed for the determination of the primary and secondary metabolites from *Mahonia aquifolium* stem bark without prior isolation. An orthogonal partial least squares to latent structures (OPLS) multivariate analysis helped correlate the chemical composition of the plant extracts to their cytotoxic activity against a human cervical adenocarcinoma cell line, which determined the protoberberine alkaloids, palmatine (**23**) and berberine (**24**); and the bisbenzylisoquinoline alkaloid, berbamine (**25**); to be powerful cytotoxic agents [77]. An interesting integrative approach that combined different omics techniques, metabolic profiling, and a biological assay significantly contributed to the study of *Oldenlandia corymbose*, a medicinal plant used in traditional medicine for cancer treatment. *Oldenlandia corymbose* was subjected to different stress conditions to investigate the impact of abiotic stress on the production of antitumor metabolites. This approach helped fill the gaps between determining the active metabolites and uncovering their biosynthetic pathway and their anticancer mechanism of action. The results showed that ursolic acid (**26**) was the major compound responsible for the anticancer activity of the extract against breast cancer cell lines. However, other compounds including leanolic acid, lutein, phytol, and pheophorbide, showed only minor contributions to this activity. It was also revealed that ursolic acid affects cancer cells mitosis [78]. In addition, the phytochemical profiling of the active root extract of *Picrorhiza kurroa* was performed using gas chromatography–mass spectrometry (GC-MS). Its metabolites were characterized by high-resolution atmospheric pressure chemical ionization mass spectrometry (HR-APCI-MS) based on high-pressure liquid chromatography (HPLC) fractionation. A compound known as Dihydromikanolide (**27**) was purified and was proposed as a promising natural compound having anticancer activity against ovarian cancer [79]. Selected examples of anticancer plant metabolites identified by metabolomics studies are presented in Table 1. Notably, the main contribution of metabolomics to these studies was the phytochemical profiling of the secondary metabolites found in the plant extracts, which was crucial for determining the biological and pharmacological actions of these plants. Furthermore, it helped the researchers in selecting target metabolites among the various metabolites identified. Undeniably, metabolomics profiling is essential besides any omic study, since it targets the end products of metabolism, which more accurately reflect the state of the organism [80]. 

Markedly, combining metabolomics and other omics studies offers several advantages. For example, an integrative study combining transcriptomic, proteomic, and metabolomic analyses revealed important findings concerning the regulatory pathways of *Prunella vulgaris* L. during development [81]. In plant research, the integration of metabolomics and transcriptomics has facilitated the discovery of relationships between genotype and phenotype through the identification of genes’ function, which controls the entire biological system in response to external influences [82]. Metabolomics studies have also been combined with in silico analyses to determine anticancer secondary metabolites. For instance, a metabolomics analysis based on liquid chromatography coupled with high-resolution mass spectrometry (LC-HRMS) allowed for the identification of the target compounds in *Curcuma longa* L and *Cosmos caudatus* extracts, curcumin (**10**) and lutein (**11**). Then, the in silico molecular docking of caspase-8 protein to these compounds revealed different types of ligand–protein interactions, suggesting these two compounds as anticancer agents [83]. In another study, metabolomic fingerprinting based on the use of reversed-phase liquid chromatography coupled to a high-resolution mass spectrometer (RPLC-HRMS) was combined with chemometric analyses and in vitro cytotoxic assays of different cancer cell lines to explore the biological potential of *Spondias mombin* and *Spondias tuberosa* plants, known in Brazilian folk medicine. RPLC-HRMS analysis and multivariate analysis allowed researchers to distinguish between the metabolomics profiles of both plant species, and helped them identify novel metabolites that were described for the first time in these species. The correlation between specific biomarkers that were supposed to be responsible for different degrees of cytotoxicity was also determined. This correlation was further validated by an in silico analysis compared to a commercial drug [84]. Another interesting point about integrated metabolomics is that any change in the level of metabolites might reflect the up- or down-regulation of the underlying biological processes [85]. These changes can be directly related to the functioning of genes that are metabolites regulators [86]. Also, comparative metabolomics studies permit the discrimination between plant species by comparing different plants’ extracts [87]. Metabolomics also provides an idea about the concentration of metabolites, allowing for both the qualitative and quantitative identification of target plants’ metabolites [88,89,90]. Interestingly, metabolomics not only permits the screening of plant metabolites [91], but also allows for the determination of the impact of medicinal plants on cancer cells following treatment [92]. It also allows the prediction of drug–target relationships through determining which signaling processes are targeted by which molecules [93]. This helps in screening the effectiveness of a therapy on cancer cells and its possible side effects. 

**Table 1:** Examples of plant metabolomics studies and their main findings in relation to cancer research. NS: not specified.

Plant	Method Used	Number of Identified Metabolites	Metabolites Classes	Cancer Cell Lines Used	Metabolites Related to the Anticancer Activity of the Extract	Ref.
*Ammi visnaga* L. (roots)	High-performance liquid chromatography–heated electrospray ionization source–high-resolution mass spectrometry metabolic profiling (HPLC-HESI-HRMS)	Several	Phenylpropanoids, flavonoids, isobenzofuranones, coumarins, chromones	Colon cancer (Caco-2), breast cancer (Mcf-7), hepatocellular carcinoma (HepG-2) cell lines	Junipediol A 4-O-glucoside (**1**), Junipediol A 8-O-glucoside (**2**), Acacetin (**3**), Apiumetin-O-glucoside (**4**). (These compounds have a possible contribution to the antiproliferative activity of the plant extract as EGFR inhibitors)	[70]
*Annona muricata* L.	Metabolomic analysis using liquid chromatography with tandem mass spectrometry (LC-MS/MS) analysis	NS	Flavonoids, steroids, sugars, alkaloids, tannins, phenols, indoles	Human lung carcinoma cell line (A549)	NS	[94]
*Antidesma bunius* L. (leaves)			Flavonoids, steroids, sugars, alkaloids, tannins, phenols, indoles, coumarins, anthrones, anthraquinones			
*Cannabis sativa* (leaves)	Untargeted metabolomic study using liquid chromatography–quadrupole time-of-flight mass spectrometry (LC-QTOF-MS)	38 (positive ionization mode) 41 (negative ionization mode)	N-containing products, polyphenols, phenylpropanoids, flavonoids, fatty acids derivates, terpenes	Gastric adenocarcinoma (AGS), melanoma (A375), human lung carcinoma (A549) cell lines	NS	[95]
Chamomile (European) flower	Metabolomic study using high-performance liquid chromatography–mass spectrometry (HPLC-MS) and NMR	Several	Phenylpropanoids, flavonoids, phenolics	Breast cancer cell line (ZR-75)	Chrysosplenetin (**4**), Apigenin. (**5**)	[71]
Chamomile (Jordanian) flower					-	
*Cissus incisa* (leaves)	Metabolomic study using ultra-high-performance liquid chromatography–quadrupole time-of-flight tandem mass spectrometry (UHPLC-QTOF-MS/MS)	171, 260, and 114 metabolites identified in different extracts	Phenolics, diterpenoids, flavonoids, fatty acid derivatives, sterols, fatty acyl, stilbene, acyl glycerol	Prostate ATCC^®^ CRL-1435 (PC3), hepatocellular ATCC^®^ HB-8064 (Hep3B), hepatocellular ATCC^®^ HB-8065 (HepG2), breast (ATCC^®^ HTB-22) MCF7, lung (ATCC^®^ CCL-185) A549, cervical ATCC^®^ CCL-2 (HeLa) cell lines	α-tocopherolquinone, phytol, grandifloric acid, cucurbitacin E, α-amyrin acetate, ursolic acid, δ-linolenic acid, oxyacanthine, stearic acid, matricin. (The cytotoxicity of the extracts might be explained by the presence of these metabolites.)	[96]
*Crocus cancellatus* subsp. *damascenus* (stigmas)	Untargeted metabolomic study using gas chromatography–mass spectrometry (GC-MS) and liquid chromatography–mass spectrometry (LC-MS)	14 (positive ionization mode) 24 (negative ionization mode)	Monoterpene glycoside, fatty acids, flavonoids	Human breast cancer cell lines (MDA-MB-231 and MCF-7)	Crocin (**6**), Crocetin (**7**), Picrocrocin (**8**), Safranal (**9**). (The antiproliferative activity of the plant extract is suggested to be due to these compounds.)	[72]
*Curcuma longa* L.	Metabolomic study using liquid chromatography–high-resolution mass spectrometry (LC-HRMS)	16	Mostly fatty acids	Activity determined in silico	Curcumin (**10**).	[83]
*Cosmos caudatus*		13			Lutein (**11**).	
*Dianthus* *caryophyllus* (different colors of carnation flower)	Targeted metabolomic study using liquid chromatography with tandem mass spectrometry (LC-MS/MS)	932	Organic acids, phenolic acids, nucleotides, flavonoids, lipids saccharides, alcohols, nucleotides and derivatives, amino acids and derivatives	Osteosarcoma (U_2_OS), human lung carcinoma (A549) cell lines	2’-Deoxyguanosine (**12**), 6-Hydroxykaempferol-3,6-O-diglucoside (**13**), Quercetin-3-O-sophoroside (**14**). (The combination of 2’-deoxyguanosine, 6-hydroxykaempferol-3,6-O-diglucoside, or quercetin-3-O-sophoroside increased antitumor activity of 2’-deoxyguanosine.)	[73]
*Dillenia* *suffruticosa* (different organs)	Genomics, transcriptomics, and ultra-performance liquid chromatography–tandem mass spectrometry (UPLC-MS/MS) analysis-based metabolomic study	Leaf (151), flower (134), root (134), stem (137)	Phenolics, alkaloids, flavonoids, terpenoids, lipids, nucleosides, amino acids, organic compounds	Cholangiocarcinoma (CCA), hepatocellular carcinoma (HCC), clear cell renal cell carcinoma (ccRCC), gastric cancer, colon cancer, prostate cancer, breast cancer, lung cancer, natural killer T cell Page 8/26 lymphoma (NKTL), diffused large B-cell lymphoma (DLBCL)	The root extract contains high levels of triterpenoids (including ursolic acid), which is known to have antiproliferative effects.	[97]
*Eleusine indica* (roots)	Ultra-high-performance liquid chromatography coupled with high-resolution mass spectrometry (UHPLC-HRMS) analysis-based metabolomic study	NS	NS	Non-small-cell lung carcinoma (H1299), breast adenocarcinoma (MCF-7), liver adenocarcinoma (SK-HEP-1) cell lines	NS	[98]
*Astragalus* *boeticus* (leaves)	1H NMR (proton nuclear magnetic resonance) and 2D (two-dimensional) nuclear magnetic resonance spectroscopy-based metabolomic study	31	Amino acids, organic acids, sugars, flavonoids, phenols, cinnamic acid derivatives, caffeic acid	Colon cancer cell lines (Caco-2, HT-29, HCT-116)	Cycloartane glycoside (6-O-acetyl-3-O-β-D xylopiranosylcycloastragenol) (**15**).	[74]
*Trigonella* *esculenta* (leaves)					Protodioscin derivative (25 R)-furost-5-ene-3β,22α,26-triol 3-O-α-L-rhamnopyranosyl-(1 → 4)-α-L-rhamnopyranosyl-(1 → 4)-[α-L-rhamnopyranosyl-(1 → 2)]-β-D-glucopyranosyl 26-O-β-D-glucopyranoside (**16**).	
*Glochidion* *velutinum* (leaves)	Liquid chromatography– tandem mass spectrometry (LC-MS/MS) analysis- based metabolomic study	48	Benzoic acid derivatives, flavans, flavones, O-methylated flavonoids, flavonoid O- and C-glycosides, pyranocoumarins, hydrolysable tannins, carbohydrate conjugates, fatty acids, coumarin glycosides, monoterpenoids, diterpenoids, terpene glycosides	Prostate cancer (PC-3), breast cancer (MCF-7) cell lines	Epigallocatechin gallate (**17**), isovitexin (**18**), ellagic acid (**19**), rutin (**20**).	[75]
Grapefruit (*C. paradisi*)	Nontargeted gas chromatography–mass chromatography (GC-MS) analysis-based metabolomic study	Several	Organic compounds (amino acids and derivatives, carbohydrates and derivatives), organic acids	Human melanoma cell line (A375)	NS.	[89]
*Kigelia africana* (fruit)	Nontargeted HPLC coupled to high-resolution time-of-flight (TOF) mass spectroscopy-based metabolomic study	356	Alkaloids, flavanoids, tannins, phenolics	Jeg-3 choriocarcinoma cell line	NS.	[91]
*Manilkara zapota* (leaves)	Liquid chromatography–tandem mass spectrometry (LC-MS/MS) analysis-based metabolomic study	NS	Flavonoids, steroids, sugars, anthraquinones, anthrones, coumarins phenols, tannins	Human adenocarcinoma cell line (A549)	NS.	[99]
*Lansium* *domesticum* (leaves)			Flavonoids, steroids, sugars, anthraquinones, indoles, triterpenes, sterols			
Lime peel (*Citrus* *aurantifolia*)	Metabolomic study using liquid chromatography– quadrupole time-of-flight mass spectrometry (LC-QTOF-MS) and gas chromatography–high-resolution mass spectrometry (GC-HRMS)	62 (detected by LC-MS) 22 (detected by GC-MS)	Glycosides, saccharides, amino acids, organic acids, alkaloids, flavonoids, flavonoids glycosides, furanocoumarins, terpenoids	Liver cancer cell lines (PLC/PRF/5)	Hesperidin (**21**), limonin (**22**), and other phytochemical components (synergistic effect).	[76]
*Mahonia* *aquifolium*	Proton nuclear magnetic resonance (1H NMR) spectroscopy-based metabolomic study	Several	Sugars, unsaturated fatty acids, protoberberine-type, aporphine-type and bisbenzylisoquinoline-type alkaloids.	Human cervical adenocarcinoma cell line (HeLa)	Palmatine (**23**), berberine (**24**), berbamine (**25**).	[77]
*Myracrodruon urundeuva* (bark, branch, and leaf)	Ultra-high-performance liquid chromatography with quadrupole time-of-flight mass spectrometry (UPLC-QTOF-MS) analysis-based metabolomic study	50	Flavonoids, phenols, tannins, quercetin derivatives, anacardic acids	Colorectal (HCT-116), glioblastoma (SF-295), leukemia (HL-60), leukemia (RAJI) cell lines	Compounds derived from quercetin, galloy derivatives, and phenolic acids (Might contribute to the high cytotoxic activity of the extracts). Quercetin derivatives, corilagin, chlorogenic acid (are known to have antitumor activity),	[100]
*Oenothera rosea*	Liquid chromatography–mass spectrometry (LC-MS) analysis-based metabolomic study	307	Organic compounds, terpenes, lipids, flavonoids	Human prostate cancer cell line (DU145)	40 metabolites were identified for having anticancer and/or antiproliferative activity.	[101]
*Oldenlandia* *corymbose* (roots, flowers, stems, and leaves)	Genomic, transcriptomic, and metabolomic study using liquid chromatography with tandem mass spectrometry (LC-MS/MS)	NS	NS	Breast cancer (SK-BR3) cell line	Ursolic acid (**26**).	[78]
*Picrorhiza kurroa* (roots)	Gas chromatography–mass spectrometry (GC-MS) analysis-based metabolomic study and high-resolution atmospheric pressure chemical ionization mass spectroscopy (HR-APCI-MS) characterization	Several	Sesquiterpenoid, alkaloids, fatty ester, others	Breast cancer (MCF7, MDA-MB-231, SKBR3), ovarian cancer (SKOV3) cell lines	Dihydromikanolide (**27**).	[79]
*Plicosepalus* *curviflorus*	Metabolomic profiling using liquid chromatography–electrospray ionization–quadrupole time-of-flight tandem mass spectrometry (LC-ESI-TOF-MS/MS)	NS	Phenolic compounds (flavonoid derivatives), triterpenes, sterols	Lung (A549), prostate (PC-3), ovarian (A2780), breast (MDA-MB-231) cancer cell lines	NS.	[102]
*Xanthium* *Strumarium* (root)	Proton nuclear magnetic resonance (1H NMR) spectroscopy-based metabolomics	Several	NS	Human ovarian cancer cell line (A2780cp)	NS.	[103]

## 3. Plant Secondary Metabolites Used for Cancer Therapy

**Figure 1:**
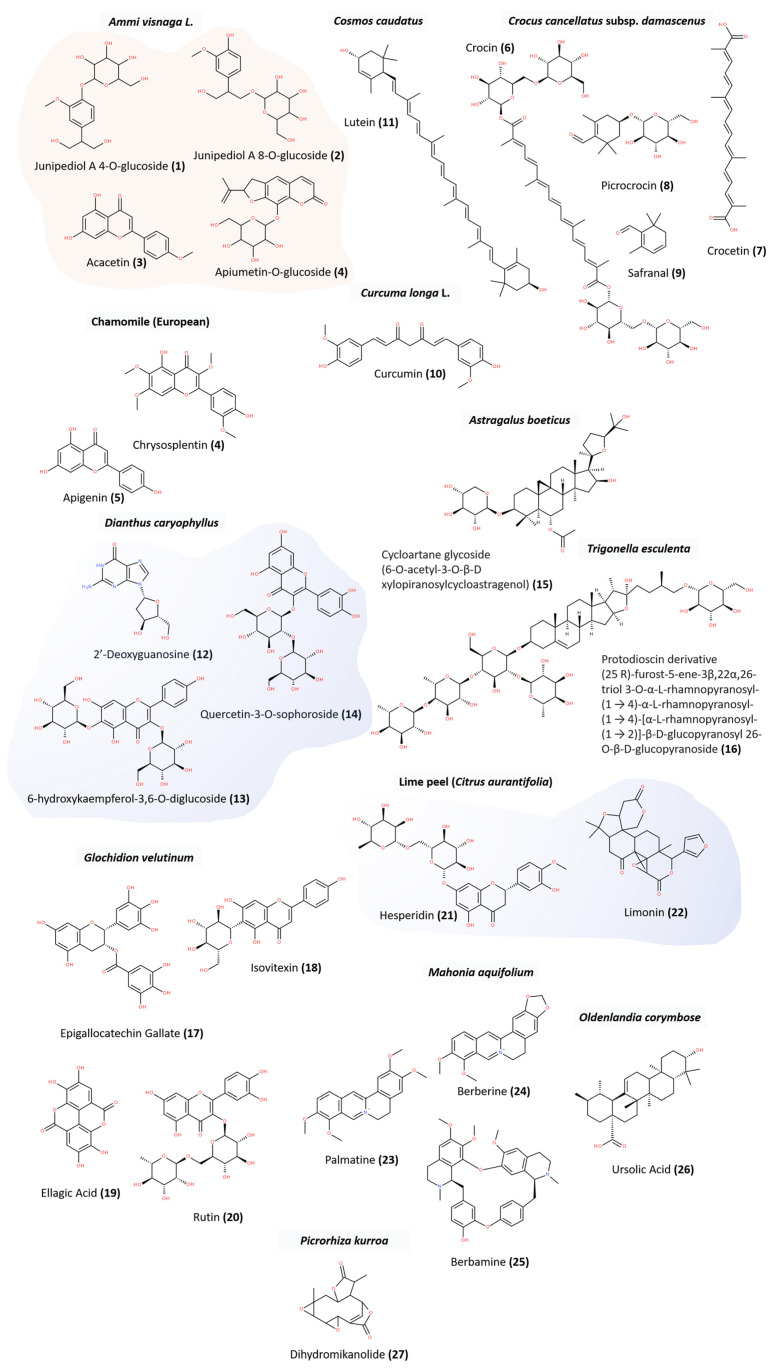
Structure of plant secondary metabolites identified by metabolomics studies reviewed in this paper. Anticancer activity exerted by these compounds is due to individual or synergistic effects. Compounds highlighted in blue are involved in anticancer synergistic effects. Compounds highlighted in pink are suggested to be related to anticancer activity of extract.

Plants contain a large number of secondary metabolites that can be classified, according to their biosynthetic origin into different categories: (i) terpenes (or isoprenoids, biosynthesized by the mevalonic acid pathway), (ii) phenolics (compounds possessing aromatic rings with attached hydroxyl groups, biosynthesized by the shikimate pathways), (iii) alkaloids (nitrogen-containing compounds other than proteins, biosynthesized from amino acids and well known for their use in medicine) and sulfur-containing compounds [104]. In fact, the antitumoral effects of medicinal plants are linked to different classes of secondary metabolites. Important medicinal plants commonly used in traditional medicine, in particular for cancer treatment, their active metabolites, and their anticancer mechanisms of action are nicely reviewed in [105]. Interestingly, some of these plants have been targeted by metabolomics studies, including *Curcoma Longa* [106], *Garcinia oblongifolia* [107], and *Perilla frutescens* [108]. Also, the major phytochemicals from different plant varieties and their molecular targets in lung cancer can be found in [109,110]. Another review also focused on the secondary metabolites that were isolated from medicinal plants traditionally used in South Africa for cancer treatment [111]. Plant secondary metabolites are the basis for the synthesis of semisynthetic derivatives used in clinical oncology [112]. These metabolites have different mechanisms of action, in which they act either as cytotoxic chemicals or as inhibitors of specific targets, such as transporters or metabolic enzymes [113,114]. Also, they show various activities, including antiproliferative [115], antiinflammatory, cytotoxic [116], and antioxidant [117]. In addition, they induce apoptosis [118] and reactive oxygen species (ROS); suppress the oncogenicity of cancer cells; and inhibit metastasis, migration, and invasion [119]. The structure of some of the metabolites identified in the metabolomics studies mentioned in this review is given in Figure 1. 

The compounds including cycloartane glycoside (6-O-acetyl-3-O-β-D xylopiranosylcycloastragenol), protodioscin derivative (25 R)-furost-5-ene-3β,22α,26-triol 3-O-α-L-rhamnopyranosyl-(1 → 4)-α-L-rhamnopyranosyl-(1 → 4)-[α-L-rhamnopyranosyl-(1 → 2)]-β-D-glucopyranosyl 26-O-β-D-glucopyranoside [74], berberine, berbamine, palmatine [77], Junipediol A 4-O-glucoside, and Junipediol A 8-O-glucoside [70], were drawn using Biovia Draw 2022. The SMILES string of all the other compounds were obtained from PubChem and their chemical structures were generated using Biovia Draw 2022 (accessed March/September 2024). The structures were saved in Mol format and then converted to SDF format.

## 4. Metabolomics as a Powerful Tool for Cancer Diagnosis and Therapy

**Figure 2:**
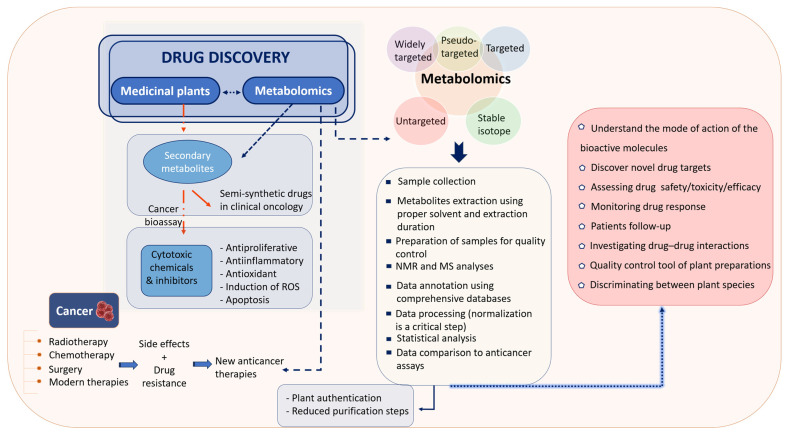
Plant metabolomics in drug discovery: concept, process, and outcomes.

Cellular abnormalities induced by cancer affect cell metabolism in different ways. These abnormalities encompass several metabolic pathways, such as carbohydrates [120], lipids [121], amino acids [122], and nucleotides pathways [123]. Metabolomics has made a significant contribution to resolving these issues through various approaches. Indeed, metabolomics is considered as a significant tool for the determination of cancer signatures that mark the early stages of the disease, allowing for the identification of new anticancer agents. These biomarkers can be found, for instance, in the plasma of cancer patients that are in the early stages of the disease [124]. So, metabolomics reveals novel diagnostic cancer biomarkers based on the variations in the metabolic profiles between patients and healthy individuals, providing insights into the altered pathways resulting from cancer-induced metabolic reprogramming. This aids in improving the survival rate and helps in the discovery of better remedies and prevention measures [125]. Also, metabolomics is employed for assessing drug toxicity. For instance, NMR-based metabolomics combined with pattern recognition data successfully identified the toxicity of Emodin, an anticancer agent found in herbal treatments, by comparing the metabolic profiles of cells before and after treatment. This demonstrated varying levels of metabolites associated with particular pathways [34]. Moreover, metabolomics is used for discovering new biomarkers that are useful for developing novel therapeutic targets. For instance, in the study by Lina A. Dahabiyeh et al., metabolomics improved the understanding of the metabolic alterations that occurred upon the treatment of cancer cells with dihydroquinazolinone derivatives, and also helped to understand the underlying mechanisms of action of the antiproliferative effects of the tested compounds [126]. It also allows to propose novel therapeutic pathways based on modifications in the concentration of specific metabolites, revealing the altered metabolic pathways that cause cancer [31]. In addition, metabolomics permits to follow-up patients at the clinical level [127]. Another interesting point is that metabolomics permits researchers to overcome the difficulties arising from the fact that the bioactivity of natural compounds does not come from the action of a single compound, but from various compounds that act synergistically [128]. Metabolomics has also improved our understanding of the chemopreventive effects of natural phytochemicals. For example, the analysis of mice serum samples by metabolomics after treatment with American ginseng extract revealed altered metabolites that were associated with inflammatory and oxidative properties of the extract; and that were responsible for its chemopreventive effect [129]. Indeed, when aiming to perform a metabolomics study, it is important to maintain the integrity of the sample before and after extraction in order to protect the metabolites from modifications, such as degradation [130]. Metabolomics uses samples from serum, plasma [131], and urine [132], thus, facilitating the conduction of high-throughput large-scale metabolomics studies [133] and making them non-invasive [134]. On the other hand, highly promising branches of metabolomics have evolved including pharmacometabonomics, which allows to develop an individualized therapy response [135]. Significantly, personalized medicine helps in the development of an effective therapy for each patient, taking into consideration their genetic profile and disease history [136]. In addition, single-cell-based metabolomics has aided in the study of cellular behavior, as well as the identification of complex cellular metabolites and the understanding of cellular and subcellular processes [137]. Therefore, progress in metabolomics is highly expected to contribute to drug development starting from an optimized diagnosis [138], passing through drug discovery [139], and ending by testing the treatment efficacy [140]. Figure 2 provides an overview of the use of metabolomics in plant-based drug discovery.

## 5. Metabolomics Approaches to Cancer Research: Untargeted, Targeted, and Beyond

Metabolomics studies are divided into different types, including untargeted, targeted [141], semitargeted, pseudotargeted [142], widely targeted [143], and stable isotope resolved metabolomics [144]. Untargeted metabolomics allows for an extensive and comprehensive analysis of a large number of unknown analytes in a sample using high-resolution mass spectrometry. Given that untargeted metabolomics allows for the development of an inclusive overview of all the metabolites present in the biological sample under study, it should be first applied prior to targeted metabolomics [141]. Untargeted metabolomics helps to determine the synergistic effects and the underlying mechanisms of drugs used in combination [145] and permits to identify novel bioactive compounds; whereas targeted metabolomics permits to measure groups of metabolites that are chemically characterized or biochemically annotated. Untargeted metabolomics relies on specific sets of known metabolites that are associated with a particular pathway and it involves the use of internal standards making the analysis semi-quantitative or quantitative [141]. Widely targeted metabolomics allows for the qualitative and semiquantitative identification of many metabolites by combining the precision of targeted metabolomics with the comprehensive analysis of untargeted metabolomics. It uses LC-MS or GC-MS or a combination of both techniques [146,147]. While, pseudotargeted metabolomics depends on the selection of a targeted ion pair, which is measured using a triple quadrupole MS by multiple reaction monitoring (MRM). An interesting feature of this technique is that it combines the advantages of both targeted and untargeted metabolomics [148]. In fact, metabolomics studies combined with in vitro cancer bioassays have helped to reduce the time required for plant-based drug discovery by correlating the data obtained from both methods [74]. In other words, the cytotoxic activity of the plant extract is measured against a specific type of cancer cell line using, for instance, MTT cytotoxicity assay, followed by subjecting the active extract fractions to metabolomics profiling in order to determine the bioactive metabolites [94]. Here are some examples highlighting the contribution of plant metabolomics to cancer research. For instance, NMR based-metabolomics study was applied to study the effect of curcumin obtained from *Curcuma longa* on the metabolome of breast cancer cells. In this study, different doses of curcumin were applied to cancer cell cultures, followed by metabolomic profiling that emphasized the dose–effect relationship of natural bioactive compounds in cancer studies [149]. In another study, an untargeted metabolomic analysis was carried out to investigate the potential of *Cissus incisa* leaves extract on cancer cells, which led to the identification of different metabolites and metabolomic pathways, with an emphasis on particular metabolites that were previously reported to have cytotoxic activity against hepatocellular cancer cells [96]. Other studies showing the contribution of plant metabolomics to cancer research and their main findings concerning the metabolic alterations identified upon treatment with different plant extracts are summarized in Table 1 and Table 2, respectively.

## 6. Plant Metabolomics Facing Challenges of Anticancer Drugs Development

The discovery of new bioactive compounds from plants is associated with several difficulties [152] that make the development of plant-based drugs challenging. Finding the precise mechanisms of action of the bioactive compounds is one of the primary causes of these difficulties [40]. Another concern is plant toxicity, which is due to the inherent toxicity of the metabolites or microbial contamination that threatens human safety [153]. Metabolomics has contributed significantly to our understanding of the mechanisms of action of diseases [154] and of drugs, thanks to the identification of the metabolites and metabolic pathways that are directly or indirectly controlled by the drug, including those mediated by the gut microbiota [155]. Also, metabolomics has enabled the evaluation of the effectiveness of plant preparations and their safety [156], which constitutes an important factor that must be tested at the pre-clinical level prior to their approval for human use. Indeed, exposure to drugs causes changes in the metabolome, such as the accumulation of drug and metabolites; in addition to alterations in the metabolites of the host’s microbiota and endobiotic. So, drug safety evaluation should be performed for both the biological sample and the target organs. Notably, the analysis of metabolic changes enables the identification of relationships between the altered metabolites, the associated pathways, and the cytotoxic mechanism. Hence, providing information about drug off-targets and allowing to understand drug toxicity [157,158]. Moreover, plant medicines are made up of a complex mixture of bioactive compounds that when introduced into the human body react with a complex system rendering the identification of drugs’ molecular targets difficult. This further complicates plant-based drug development. Interestingly, metabolomics helps to find molecular targets of medicinal plants by elucidating the changes affecting the endogenous metabolites’ behavior following drug administration [159]. Also, pharmacometabolomics permits to predict drug response through the identification of the altered metabolites or the dysregulated metabolic pathways [160]. Knowing that different types of cancer show resistance to chemotherapies [161], researchers have developed multitargeted therapies as promising tools to enhance the pharmacological potency of anticancer drugs [162]. In this context, metabolomics has helped reveal the multitarget effects of plants against cancer cell lines’ growth by targeting key metabolic pathways [163]. Metabolomics, in integration with other omics studies, has also allowed the identification of crucial metabolites, paving the way for the prediction of drugs concentration in the treated organ. This facilitates the understanding of drug bioavailability and clearance [164]. Furthermore, metabolomics has emerged as a promising tool for quality control of plant medicines using different analytical techniques; mainly, NMR-based strategies. Indeed, quality control of plant preparations is necessary for ensuring the reproducibility of the therapeutic activity of the active compounds found in different batches [165,166]. It also serves as a tool for the authentication of plant-based products based on specific biomarkers that discriminate between close plant species [167]. In fact, plant authentication is one of the most significant stages of plant-based drug discovery. Therefore, it is crucial to authenticate the plant species from which the compounds were isolated in order to correctly associate the compounds’ therapeutic benefits to the appropriate plant species. This will facilitate further research on the identified compounds in the future [168]. In addition, metabolomics can be efficiently used to discriminate between plant samples prepared under different conditions based on metabolic variations [169].

## 7. Avoiding Difficulties and Performing Successful Plant Metabolomic Analyses

Metabolomics is a highly promising tool for cancer research [170]. However, in order to obtain reliable results, certain measures need to be taken into account at every stage, from sample preparation to data analysis [171]. It is worth noting that achieving a comprehensive profiling of all metabolites using a single method is not possible. Herein, we provide a list of several key steps to be considered and the errors to be avoided when aiming to conduct a successful metabolomics analysis of anticancer medicinal plants, Table 3.

## 8. Challenges of Plant Metabolomics-Based Anticancer Drug Development and Possible Solutions

Although metabolomics studies are highly promising, they are associated with a number of difficulties, such as analytical and technical challenges [200,201]. Accordingly, it is critical to search for optimal extraction methods and to standardize protocols in order to ensure the proper data exchange between laboratories and to establish uniform databases [173]. One of the major problems associated with metabolomics is that performing metabolomic analyses solely in vitro may lead to incomplete results, which will not reflect natural cell behavior. This problem can be solved by the combination of both in vivo and in vitro studies, which allows for a more reliable study of cancer metabolism [202]. Another difficulty is the samples’ incompatibility with most of the analytical techniques used for metabolomics analyses. This might lead to certain losses in the poorly fixed metabolites from the liquid chromatography (LC) column in the hyphenated MS system, which could lead to insufficient ionization during mass spectrometry [203,204]. Also, some metabolites are not suitable for metabolomics applications, since they can degrade easily throughout the extraction process [202]. Although NMR is an excellent tool for metabolomics research, there are a number of challenges associated with the identification, quantitation, and reliable detection of metabolites by this technique [205]. Moreover, great attention should be given to the statistical approaches used in metabolomics studies, in particular, the method, the number of samples, and the duplication of studies [206,207]. On the other hand, thousands of metabolites (or mass peaks) have been detected using untargeted metabolomics experiments, but the main problem is identifying these metabolites. Indeed, metabolite annotation is a critical step to achieve the reliable identification of metabolites. But it constitutes a major difficulty, especially for untargeted metabolomics [208,209]. The challenge of acquiring a comprehensive phytochemical profile is demonstrated by a study that sought to determine the anticancer activity of *Kigelia africana* extracts; only 63.8% of the detected signals were identified after a comparison with the available data libraries. Thus, the lack of inclusive databases leads to the incomplete identification of the detected signals, and complicates the differentiation between isoforms. This limits the identification of all the compounds that are responsible for the anticancer activity of the extract [91]. Nevertheless, some studies have developed new strategies to overcome the limitations of databases. For instance, a structural motif-based approach (SUMMIT Motif), was developed to identify the undiscovered metabolites, in which their spectroscopic signatures have no matches in the available metabolomics databases. This approach requires no extensive purification, and uses NMR in tandem with new NMR molecular structural motif metabolomics databases [210]. Another study used a novel strategy called structure of unknown metabolomic mixture components by MS/NMR (SUMMIT MS/NMR), which allowed for the identification of unknown metabolites without any purification, and with no need for MS and NMR databases [191]. Also, several attempts have been carried out to improve metabolomic annotation [211,212]. For example, the use of tandem mass spectrometry reveals additional structural data leading to better annotation [194]. There exist several open databases that can be used for natural product identification, including MassBank [213] and Metlin databases [214], in addition to PRIMe [215] and PlantMetSuite, which are public website databases [216]. Another commercial database is Wiley Registry/NIST Mass Spectral Library that provides electron ionization (EI) and tandem MS/MS spectra [217]. Also, in-house spectral libraries have been constructed and used for plant studies [216,218], but they are not accessible by other laboratories. Notably, there are around two thousand standards available for expanding plant metabolites spectral libraries [219]. While metabolomics studies help identify phytochemicals and, thus, eliminate the need for long purification steps [220], one should take into account to what extent metabolomics data can be considered reliable and reproducible [172]; as this might affect the accuracy of the link established between the health benefits of different plants and their phytochemicals’ content.

## 9. Conclusions

Conducting fruitful cancer research and finding novel therapeutics necessitates a close investigation of the changes impacting the biochemical state and the underlying biological processes occurring in cells, tissues, and organisms. This can be only achieved through well-planned metabolomics research, which is predicated on rapid, comprehensive, and reliable identification of metabolites. Metabolomics studies give insights into the metabolic network rearrangements that naturally occur during the transformation of normal cells into malignant cells. So, discovering these metabolic changes through a comprehensive identification of metabolites, will lead to a better understanding of the mechanisms of action of anticancer drugs, and to the identification of novel biomarkers. In this regard, finding good correlations between metabolites obtained from both the medicinal plants and the biological samples taken from patients will help develop new effective therapies. Also, a critical point to consider is the patients’ backgrounds and the external factors, which can greatly affect metabolic profiling. Since metabolomics is still in the developmental stage, it faces several difficulties. The lack of effective strategies for the identification of metabolites, the need for a standardized method, the low detection sensitivity of the analytical techniques, the difficulty in achieving unbiased and high-throughput quantitative analyses, and the absence of comprehensive databases constitute the major problems for drug discovery from medicinal plants. Combining NMR and MS techniques in addition to the integration of metabolomics with other omics studies remains indispensable for metabolomics to uncover the different mechanisms and the underlying pathways. All in all, a profound knowledge of metabolomics in plants and in the human body can help better comprehend the body response to therapy, making it possible to determine the optimal dose, and to develop individualized treatment. Also, discovering biomarkers for cancer diagnosis holds great promises for detecting the early stages of the disease and save patients a lot of pain and suffering. The main purpose of this work was to guide researchers to develop well-designed metabolomics studies for cancer research. In the first place, we emphasized the importance of metabolomics in the search for plant-based therapeutics. Furthermore, we detailed several medicinal plants and the different metabolomics approaches used to identify anticancer metabolites. Then, we outlined the most important parameters that researchers should consider before conducting any metabolomics research, as well as the potential advantages. We also reviewed the most common challenges that researchers might face when aiming to perform metabolomics studies, and we provided some solutions. 

## Figures and Tables

**Table 2:** Examples of some plants and their main cancer inhibition pathways.

Plant	Altered Metabolic Pathway	Cancer Type	Study
*Aloe vera* (leaves)	Protein biosynthesis, catecholamine biosynthesis, mitochondria transport chain, and pentose phosphate pathway.	Raji cell lines (cancerous lymphoma cells)	[150]
American ginseng (*Panax quinquefolius* L.)	Amino acid, lipids, and carbohydrates metabolism. Metabolites involved in inflammation and oxidation.	Colon carcinogenesis	[129]
*Xanthium strumarium*	Tyrosine metabolism, nucleotide metabolism, fatty acid biosynthesis, and glycerolipid metabolism.	Ovarian cancer cell line (A2780cp)	[103]
*Xanthium strumarium*	Aminoacyl-tRNA synthesis, glycerolipid metabolism, fatty acid biosynthesis, and biotin metabolism.	Epithelial ovarian cancer cell line (SK-OV-3)	[151]

**Table 3:** Illustration of the key points for designing an effective metabolomics study for the investigation of medicinal plants in cancer research.

Procedure standardization	To consider	The metabolomics procedure including sample preparation, measurement and data analysis should be standardized [67].
	Benefit	Standardization of all the steps facilitates the direct comparison of data obtained in different laboratories (inter/intra-laboratory exchanges). This helps to ensure reproducibility [172], and broaden the identification of key metabolites; it also permits the building of comprehensive metabolomics databases [173].
Plant collection, sample size, preparation and extraction	To consider	- Plant samples should be quickly collected [174]. - The season and the tissue from which the bioactive compounds are to be isolated should be carefully chosen [20,175].
	Benefit	- Avoiding activity loss and change in metabolites’ composition [130,175,176]. - Obtaining the highest activity of metabolites.
	To consider	Samples should be immediately frozen in liquid nitrogen and stored at − 80˚ C or lyophilized.
	Benefit	Avoiding any significant changes in plant composition [20].
	To consider	The sample size should be considered.
	Benefit	Avoiding non-reliable results [177].
	To consider	Quality assurance (QA) (the measures implemented by the laboratory to ensure that quality standards will be fulfilled) and quality control (QC) samples (the quality of untargeted data is ensured by preparing various types of mixtures) should be used in untargeted metabolomics studies.
	Benefit	- Acquiring high-quality publishable data. - Avoiding batch variations [178,179,180].
	To consider	An efficient extraction method using a solvent system with the proper solvent and solvent-to-sample ratio should be used. Attention should be paid to the extraction duration.
	Benefit	An effective extraction method enables the extraction of the maximum quantity of metabolites and provides access to low abundant compounds that are difficult to extract. It also helps in achieving reproducible quantification of metabolites [20,181,182,183].
Combining metabolomics studies and anticancer activity bioassays	To consider	Metabolomics results should be compared to data from biological assays.
	Benefit	- Achieving reliable correlations between the identified phytochemicals and the biological activity of the extract. - Achieving good discrimination between the different samples based on their activity, in correlation with metabolites identification [184].
MS-based metabolomics analysis	To consider	Metabolomics approach involving experimental deconvolution of the tandem mass spectrometry (MS/MS) data acquired in a broad MS isolation window (ex. 9 Da) is recommended (For more details, see reference) [185,186]. Direct analysis of the samples using quadrupole (Q) time of flight-mass spectrometry (TOF-MS) [187] or a Fourier transform ion cyclotron MS (FT-MS) [188] or direct infusion of the samples using direct infusion mass spectrometry (DIMS) and flow infusion mass spectrometry (FIMS) is highly useful [189].
	Benefit	- The experimental deconvolution of MS/MS data acquired in a broad MS isolation window permits to obtain high quality spectra and the identification of novel metabolite [185,186]. - TOF instruments provides high mass accuracy and high resolution, and allows to detect large diversity of masses [187]. - Fourier transform ion cyclotron MS (FT-MS) eliminates any need for chromatography prior to analysis [188]. - Direct infusion mass spectrometry provides reduced instrument cycle times, reduced sample pretreatment and high-throughput screening (analysis of more than 1000 samples/week) [189].
NMR-based metabolomics analysis	To consider	The use of NMR analysis for metabolomics studies is highly recommended.
	Benefit	Quantification of metabolites [190].
Combining analytical platforms (MS-NMR)	To consider	The integration of different analytical platforms (mass spectrometry (MS) and NMR techniques) in a metabolomics study is highly recommended. [191,192].
	Benefit	Obtaining broader metabolome coverage and high-quality data using hyphenated separation platforms [191,192].
Sample analysis by MS-based targeted metabolomics	To consider	- Ultra-high-performance liquid chromatography coupled to a triple quadrupole MS (UPLCQqQ-MS), which is operated in MRM (Multiple Reaction Monitoring) mode is an ideal technique to be used in targeted metabolomics [193,194]. Attention to false positives is necessary [177]. False positives might be observed as a result of isomeric metabolites having similar product ion used to detect the target compounds, but are inadequately separated by liquid chromatography [193,194].
	Benefit	- UPLCQqQ-MS is sensitive, reproducible, characterizes a wide range of compounds, and allows for robust quantification [193,194]. - Avoiding false metabolic data [177].
	To consider	Metabolomics data standards are useful.
	Benefit	Ensure reproducible research [195].
Integrated metabolomics analysis	To consider	Metabolomics studies should be integrated with other Omics studies including genomics, proteomics, transcriptomics, and metabolomics is highly recommended.
	Benefit	- Obtaining a comprehensive picture of cancer metabolism [196]. - Revealing biosynthetic pathways and mechanisms of action of active metabolites [78].
Data normalization	To consider	Metabolomics data should be normalized.
	Benefit	- Minimizing batch-to-batch variations. - Important for large-scale metabolomics analyses [197].
Metabolomic profiling in relation to previous studies	To consider	Metabolic profiling of plants previously reported to have anticancer activity is highly useful.
	Benefit	- The generation of a database will allow for robust differentiation of active extracts and for the selection of target bioactive metabolites. - Speeding up drug discovery from anticancer plants [91].
Assessing response to treatment	To consider	Analysis of the biological sample (cells/tissues) following treatment with plant extracts/extracted plant compounds is useful.
	Benefit	- Validation of the effectiveness of the extract/extracted bioactive compounds. - Elucidation of the metabolic alterations induced in the host cell upon treatment. - Understanding the impact of the treatment on cancer metabolism [198,199].

## Data Availability

Not applicable.
